# Who and how to screen for endogenous hypercortisolism in a high-risk population: a special issue of the journal of endocrinological investigations

**DOI:** 10.1007/s40618-024-02449-5

**Published:** 2024-10-01

**Authors:** Filippo Ceccato, Massimo Terzolo, Carla Scaroni

**Affiliations:** 1 Department of Medicine DIMED, University of Padova, Padova, Italy; 2 Endocrine Disease Unit, University-Hospital of Padova, Padova, Italy; 3 Internal Medicine, Department of Clinical and Biological Sciences, San Luigi Hospital, University of Turin, Orbassano, Italy

The diagnosis of endogenous hypercortisolism is a challenge because clinicians should screen for a rare disease [1] in high-risk populations, characterized by one or more of the following diseases: diabetes, hypertension, obesity, fractures, hirsutism, mood disorders, and unusual infections. Moreover, some of these conditions are common in the general population. In particular, the simultaneous presence of some of these diseases that may cluster in the metabolic syndrome increases the chance of an underlying cortisol excess [2]. Therefore, many patients should be screened before securing a diagnosis of Cushing’s Syndrome (CS).

The recommended screening tests are the measurement of cortisol levels after dexamethasone suppression, in 24-hour urinary excretion, or at late night (usually in saliva). The result of a first-line test should be integrated with the clinical presentation of the patient. A screening test should prefer sensitivity to identify CS among the more frequently encountered cases of metabolic syndrome, obesity, and hypertension, at the cost of reduced specificity. To enhance the diagnostic conundrum, the detection of an adrenal incidentaloma is an increasingly common phenomenon in the general population (up to 10% of older adults), and 40% of patients are characterized by a mild autonomous cortisol secretion, which may contribute to the high prevalence of comorbidities in patients with adrenal incidentalomas [3, 4]. In clinical practice, a continuum of cortisol secretion goes through diabetes, obesity, and overt CS [5]. In the metanalysis of the Endocrine Society in 2020 on the screening tests for CS, the individual studies summed up to 299 participants (median 82 subjects), with a prevalence of endogenous hypercortisolism ranging from 3 to 91% (median 27%). The metanalysis concluded that all of the included diagnostic tests for endogenous hypercortisolism are highly sensitive and specific [6]. CS overrepresentation is a common bias in the studies reported in the metanalysis; moreover, one of their major drawbacks is that they reported the results of one screening test in a single high-risk population, mainly in a tertiary endocrine setting. Outside of a referral center, the prevalence of CS in high-risk populations may be remarkably lower than expected. Moreover, another common bias of these studies is the usual lack of a gold standard test to be used as a comparator. Several authors reported that routine screening in high-risk populations is not recommended in all patients [7].

The present project, endorsed by the Italian Society of Endocrinology (SIE) in 2023, aims to define who are the high-risk subjects and which is the best first-line screening test for each high-risk condition. The research question is “how to shorten the time to diagnosis in CS”. The project scheme, conceived by the Coordinators of the Pituitary and Adrenal Club of the SIE, consists in two steps: first, the publication a Special Issue of the Journal of Endocrinological Investigation including eight of state-of-the-art short reviews focusing on each of the identified high-risk conditions (arterial hypertension, osteoporosis and fractures, diabetes mellitus and obesity, pediatric conditions, mood disorders, unusual infections and thrombotic events, adrenal and pituitary incidentaloma, and woman’s health) and, second, a Position Statement that aims to suggest the best screening test, tailored to the patient’s clinical condition. The effort of the Authors of the Position Statement will be to write a practical and useful document for all physicians who visit a patient with suspected CS. A panel of Italian experts on CS has been selected and invited to write reviews that summarize the current knowledge regarding CS screening in the aforementioned eight high-risk conditions, offering us their vast experience for a timely and scholarly Special Issue. This Special Issue of the Journal of Endocrinological Investigations collects the eight reviews, providing an up-to-date and cutting-edge volume dedicated to CS screening. The work of literature revision, performed by the authors, will be used by a panel of experts to write the Position Statement of the SIE regarding the screening of endogenous CS. Actually, CS is diagnosed approximately three years late [8] in a patient with a peculiar clinical picture after months/years of consultations and unnecessary biochemical or imaging studies. It is authors’ aim that the main result of the Special Issue and the Position Statement will be to increase awareness of endogenous hypercortisolism among physicians, not only endocrinologists, to reduce the gap between disease onset and final diagnosis of hypercortisolism.

## Data Availability

Not available.
