# Correction to: Who and how to screen for endogenous hypercortisolism in a highrisk population: a special issue of the journal of endocrinological investigations

**DOI:** 10.1007/s40618-025-02545-0

**Published:** 2025-03-03

**Authors:** Filippo Ceccato, Massimo Terzolo, Carla Scaroni

**Affiliations:** 1 Department of Medicine DIMED, University of Padova, Padova, Italy; 2 Endocrine Disease Unit, University-Hospital of Padova, Padova, Italy; 3 Internal Medicine, Department of Clinical and Biological Sciences, San Luigi Hospital, University of Turin, Orbassano, Italy

**Correction to: Journal of Endocrinological Investigation**

10.1007/s40618-024-02449-5

The article “Who and how to screen for endogenous hypercortisolism in a highrisk population: a special issue of the journal of endocrinological investigations”, written by Filippo Ceccato, Massimo Terzolo and Carla Scaroni, was originally published under exclusive license to © The author(s), under exclusive licence to Italian society of Endocrinology (SIE) 2024. As a result of the subsequent decision to publish the article under the open access model, the article’s copyright notice was changed on 24 January 2025 to © The author(s) 2025 and this article is licensed under a Creative Commons attribution 4.0 international license, which permits use, sharing, adaptation, distribution and reproduction in any medium or format, as long as you give appropriate credit to the original author(s) and the source, provide a link to the Creative Commons licence, and indicate if changes were made. The images or other third-party material in this article are included in the article’s Creative Commons licence, unless indicated otherwise in a credit line to the material. If material is not included in the article’s Creative Commons licence and your intended use is not permitted by statutory regulation or exceeds the permitted use, you will need to obtain permission directly from the copyright holder.

To view a copy of this licence, visit http://creativecommons.org/licenses/by/4.0/

The original article has been corrected.

