# Correction: Who and how to screen for endogenous hypercortisolism in a high-risk population: a special issue of the Journal of Endocrinological Investigation

**DOI:** 10.1007/s40618-024-02484-2

**Published:** 2025-03-12

**Authors:** Filippo Ceccato, Massimo Terzolo, Carla Scaroni

**Affiliations:** 1 Department of Medicine DIMED, University of Padova, Padova, Italy; 2 Endocrine Disease Unit, University-Hospital of Padova, Padova, Italy; 3 Internal Medicine, Department of Clinical and Biological Sciences, San Luigi Hospital, University of Turin, Orbassano, Italy

**Correction: Journal of Endocrinological Investigation ** 10.1007/s40618-024-02449-5

In the original version of this article, Some part of the Funding information was missing. The funding information was previously read "Recordati Rare Diseases" should have been "Recordati Rare Diseases - Open Access funding for this article was provided by Recordati Rare Diseases Italy S.r.l. The funder had no role in the preparation, review, or approval of the manuscript."

The original article has been corrected.

